# The Self and Its Right Insula—Differential Topography and Dynamic of Right vs. Left Insula

**DOI:** 10.3390/brainsci11101312

**Published:** 2021-10-02

**Authors:** Andrea Scalabrini, Angelika Wolman, Georg Northoff

**Affiliations:** 1 Department of Psychological, Health and Territorial Sciences (DiSPuTer), G. d’Annunzio University of Chieti-Pescara, Via dei Vestini 33, 66100 Chieti, Italy; 2 The Royal’s Institute of Mental Health Research, Brain and Mind Research Institute, Ottawa, 145 Carling Avenue, Rm. 6435, Ottawa, ON K1Z 7K4, Canada; Georg.Northoff@theroyal.ca; 3 School of Psychology, University of Ottawa, 136 Jean-Jacques Lussier, Ottawa, ON K1N 6N5, Canada; 4 Centre for Neural Dynamics, Faculty of Medicine, University of Ottawa, Roger Guindon Hall 451 Smyth Road, Ottawa, ON K1H 8M5, Canada; 5 Mental Health Centre, Zhejiang University School of Medicine, Tianmu Road 305, Hangzhou 310013, China; 6 Centre for Cognition and Brain Disorders, Hangzhou Normal University, Tianmu Road 305, Hangzhou 310013, China

**Keywords:** self, basis model of self-specificity (BMSS), right anterior insula, topography, dynamic, fMRI, EEG, resting state, autocorrelation window, degree of centrality

## Abstract

Various studies demonstrate a special role of the right compared to the left anterior insula in mediating our self. However, the neural features of the right insula that allow for its special role remain unclear. Presupposing a spatiotemporal model of self—“Basis model of self-specificity” (BMSS)—we here address the following question: what spatial-topographic and temporal-dynamic features render neural activity in the right insula to be more suitable in mediating self-specificity than the left insula? First, applying fMRI, we demonstrate that the right insula (i) exhibits higher degrees of centrality in rest, and (ii) higher context-dependent functional connectivity in a self-specific task among regions of distinct layers of self (intero-, extero-proprioceptive, and mental). Second, using EEG in rest and task, we show that the right insula shows longer autocorrelation window (ACW) in its neural activity than both left insula and other regions of the different layers of self. Together, we demonstrate special topographic, i.e., high functional connectivity, and dynamic, i.e., long ACW, neural features of the right insula compared to both left insula and other regions of the distinct layers of self. This suits neural activity in the right insula ideally for high functional integration and temporal continuity as key features of the self including its intero-, extero-proprioceptive, and mental layers.

## 1. Introduction

The self is a key feature of our mental life that allows integrating various inputs including intero-, extero-, and proprioceptive [1,2]. Besides cortical midline structures [3,4,5,6], one key region is the insula. Several studies observed the predominant involvement of the right rather than left insula during self-referential tasks or stimuli [7,8,9,10,11,12,13,14]. The key role of the right insula for self-reference has been hypothesized early on by Craig who, based on anatomical differences, suggested the right insula to preferentially mediate the self [15,16]. What are the neural features that render the right insula more suitable than the left insula to process self-specificity? Addressing this yet open question is the main goal of our study.

A recent large-scale imaging/fMRI meta-analysis by Qin et al. [17] confirmed the key role of the right insula for the self. They observed especially the right insula, together with left insula, dorsal anterior cingulate cortex, thalamus, and parahippocampus, to be involved in mediating the interoceptive self, that is, the self that is recruited during tasks requiring interoceptive awareness like the awareness of one’s own heartbeat. The role of the insula was not limited to the interoceptive self though. They also observed the right insula, together with left insula, interior frontal gryus, premotor cortex, temporo-parietal junction (TPJ), and medial prefrontal cortex (MPFC), to be recruited in other studies of self like extero- proprio-ceptive self about the outer boundaries of the own body (see also [18]). Finally, the right insula was also observed in the typical studies on mental or cognitive self-reference using trait adjectives (and related paradigms) that strongly recruit the DMN, i.e., cortical midline structure.

Together, these observations suggest that especially the right insula provides the “glue” or connection between different layers of self, interoceptive, proprioceptive, and mental by means of which it can be distinguished from familiarity. Together, these findings describe hierarchical model of self [17] showing how regions of the interoceptive self were also included in the other layers like extero-proprioceptive and mental self where they were complemented by additional regions extending the topography of the self.

The goal of our study is to address the following question: what renders neural activity in the right insula special compared to the left insula such that the former can provide the “glue” or connection between the different layers of self? For that purpose, we investigate spatial topography and temporal dynamic of right vs. left insula with respect to those regions mediating the different layers of self. Our first specific aim was to analyse spatial topography of right vs. left insula in fMRI rest and task states by a network model approach and task context dependent functional connectivity of all those regions implicated in the Qin et al. [17] meta-analysis on self. Given that the self is suggested to serve an integrative role on neural [17] and cognitive [1] grounds, we hypothesized higher degrees of centrality and context-dependent functional connectivity (as measure of functional integration during a self-related vs. non self-related task) of the right insula to the rest of the brain’s self-specific regions compared to the left insula.

Our second specific aim was to analyse the temporal features of the right vs. left insula with respect to the self. That was investigated in an EEG data set applying resting state and a morphed self-face task. We focused specifically on the Intrinsic Neural Timescales (INT; also called temporal receptive window; TRW [19]) defined as the duration of the brain to integrate information [20] and is known to mediate input processing and, more specifically temporal integration or segregation of different inputs [21,22,23]. The longer the time windows of the neural activity of a region, the more inputs at multiple distant points in time can be summed and pooled together under the umbrella of one temporal window and subsequently elicit one (rather than multiple) neural activity change, i.e., task-related activity [24].

These timewindow lengths can be quantified via the autocorrelation window (ACW). By correlating the signal with temporally shifted versions of itself, one can measure the moment when the correlation decays below a certain value, usually when r < 0.5. This threshold can be reached earlier (shorter window) or later (longer window) in time. Shorter ACW reflect faster neural fluctuations while longer ACW are linked to slower fluctuations. Hence, the ACW can give insight in the intrinsic neural timescales.

Given the role of the insula and the right insula in particular in mediating integration of intero- and exteroceptive inputs [15,16], we assume longer INT, i.e., longer ACW, in the right insula than the left insula. Intero- and exteroceptive, proprioceptive, and even internal cognitive/mental inputs may be temporally integrated to a higher degree by the longer time windows of the right insula (than the shorter ones of the left insula). This suits the right insula ideally to provide the “dynamic or temporal glue” or connection between the three layers of self [17].

## 2. Materials and Methods

Our investigation comprised different steps and methods. First, we focused on an fMRI sample to investigate the spatial topography of right vs. left insula. Secondly we focused on an EEG to investigate the temporal dynamic of right vs. left insula. Moreover, we further investigated the role of right vs. left insula in different states, that are the resting- and the task-state in both fMRI and EEG.

### 2.1. fMRI

#### 2.1.1. fMRI Sample

The fMRI sample was constituted by 32 right-handed male participants (age 21–33; mean = 25.4; standard deviation = 2.82). They are the same as those included in previous studies (for more detail see [8,9]). All participants had normal or corrected-to-normal vision capabilities. None of the participants reported a history of neurological or psychiatric disease, or substance abuse. Written informed consent was obtained from all participants after full explanation of the study procedure, in line with the Declaration of Helsinki. The Ethics Committee for Biomedical Research of the provinces of Chieti and Pescara approved the experimental protocol.

#### 2.1.2. Resting State fMRI

A total of 32 subjects completed resting state fMRI acquisition (2 resting state of 6 min each, eyes open and fixation cross).

#### 2.1.3. Task fMRI

In total, 21 out of the 32 participants (age 21–30; mean = 24.9; standard deviation = 2.45) also completed task fMRI acquisition. During the task fMRI runs (8 runs of 7.8 min each), a wooden table was placed on the participant’s legs. An inanimate target (mannequin hand) and an animate target (the hand of another volunteer who was standing next to the scanner) were both placed next to the participant’s hand. The participant completed a series of active touch and no-touch trials. Active touch was performed through an object to avoid the direct physical sensation of touching the different targets, that is, the hand of a volunteer (animate target) or a mannequin hand (inanimate target; for more details on the task see [8]). For the purpose of our investigation, we focused specifically on active touch trials and on animate and inanimate conditions. The different targets were used as a proxy of self-relatedness and non-self relatedness (respectively) since our previous findings [8] showed, at a behavioral and neuronal level, how the active touch towards the animate stimuli is featured by higher degree of self-relatedness vs. lower degree of self-relatedness as linked with the inanimate target. Thus, our task showed the intimate link between actively touch another animate target and self-relatedness.

#### 2.1.4. fMRI Data Acquisition and Preprocessing

For each participant, BOLD contrast functional imaging was performed with a Philips Achieva scanner (Andover, MA, USA) at 3 T at the Institute of Advanced Biomedical Technologies, Chieti, Italy. An initial T1-weighted anatomical (3D TFE pulse sequence) was acquired with the following parameters: field of view = 240 mm; voxel size = (1 mm × 1 mm × 1 mm); TR 8.1; TE 3.7. Two resting-state fMRI runs (number of fMRI frames/run: 180) and 8 task fMRI runs (number of fMRI frames/run: 235) were acquired in a single fMRI session (T2* weighted EPI sequence with TR = 2000 ms; TE = 35 ms; 31 slices; slice thickness = 3.5 mm; in-plane voxel size = 2.875 × 2.875; field of view = 230 mm; flip angle = 90°). Preprocessing procedures of the fMRI data were implemented in Analysis of Functional NeuroImages software (AFNI; [25]; http://afni.nimh.nih.gov/afni accessed on 28 August 2021) including: (1) slice timing correction; (2) rigid body correction/realignment within and across runs. Six head motion parameters, 3 translational and 3 rotation parameters, were estimated and frame-wise realignment was performed using AFNI’s 3dvolreg command. After the estimated motion parameters were visually inspected, participants with head motion larger than ±2 mm translation or ±2.5° rotation were eliminated [26]; (3) co-registration with high-resolution anatomical images; (4) spatial normalization into Talairach stereotactic space; (5) resampling to 3 × 3 × 3 mm^3^ voxels; and (6) regression of linear and nonlinear drift (equivalent to a high-pass filtering of 0.0067 Hz), head motion and its first-order temporal derivative, and mean time series from the white matter (WM) and cerebrospinal fluid (CSF) to control for non-neural noise [27]. The WM and CSF masks were eroded by one voxel [28] to minimize partial voluming with gray matter; (7) spatial smoothing with an 8 mm fullwidth at half-maximum isotropic Gaussian kernel.

#### 2.1.5. Definition of Regions/Nodes of Interest (ROIs)

**Figure 1:**
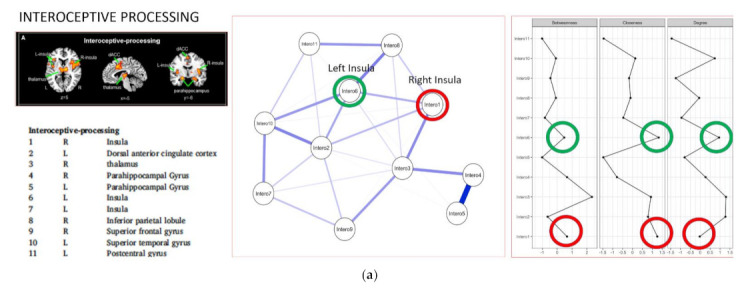

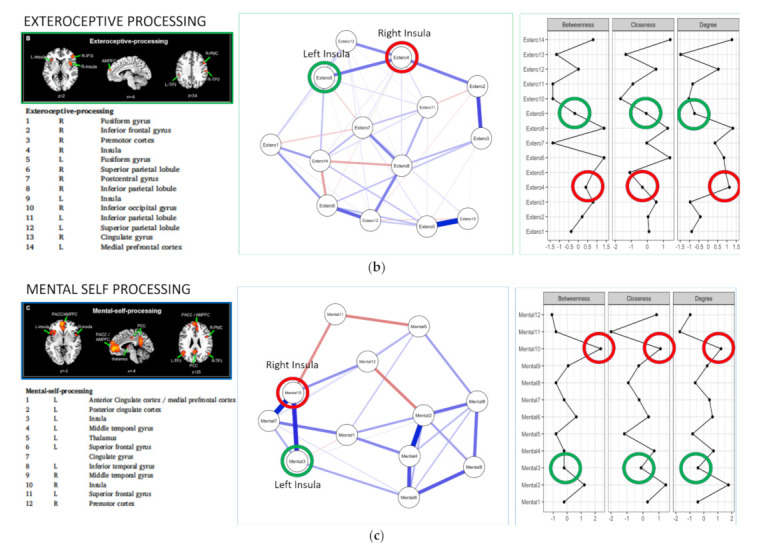
Resting state network analyses of the Three Layers Model of Self. (**a**) Interoceptive processing level; (**b**) exteroceptive processing; (**c**) mental self-processing. Blu lines indicate positive relation between nodes. Red lines indicate negative relation between nodes. The thickness of the lines indicates the degree of relation between nodes.

In order to investigate the spatial configuration of the three layers of self [17] in spontaneous activity of the brain (during resting state fMRI) regions of interest (ROIs) were taken from each layer of self of the hierarchical model proposed recently by Qin et al. [17] i.e., (a) the interoceptive self; (b) the extero- proprio-ceptive self and (c) the mental self (see Figure 1 for a list of regions included in each layer of self). All the regions were created with a 10 mm diameter (spherical) centered using the coordinates reported in [17]. According to our aim we focused especially on the role of right anterior insula (vs. the role of left anterior insula).

#### 2.1.6. Resting State Analysis

The same preprocessing steps described above were performed on resting state data with the addition of temporal band-pass filtering (0.01 < f < 0.1 Hz) to reduce low-frequency drift and high-frequency respiratory/cardiac noise [29,30]. Given the methodological issues recently raised [19,31,32], global signal regression (GSR) was not included in the preprocessing of the resting state fMRI data. In graph theory, a complex system is modelled as a “graph”, which is defined as a set of “nodes” (each ROI) linked by “edges” (regularized partial correlations between pairs of nodes controlling for all other nodes in the network). To investigate the relationship between the different regions-nodes of each layer of self [17] a network approach analysis (performed in JASP) was used in order to investigate *Closeness* (the inverse of the sum of all shortest paths from the node of interest to all other nodes), *Betweenness* (the number of shortest paths that pass through the node of interest), *Degree* (*nodal strength*: the sum of the absolute input weights of that node. In general, a higher *Degree* measure indicates that this node has a central role in the network). We used EBICglasso technique (computing a sparse gaussian graphical model with the graphical lasso [33]. Tuning parameter was set at 0.5 using the Extendend Bayesian Information criterium (EBIC) this 0.5 indicate that more parsimonious models with fewer edges are preferred—This method provides a network of partial correlation coefficients with a limited number of spurious edges [34,35]. Centrality indices are plotted using standardized z-scores in order to facilitate interpretation.

#### 2.1.7. Task Functional Connectivity—gPPI

**Figure 2:**
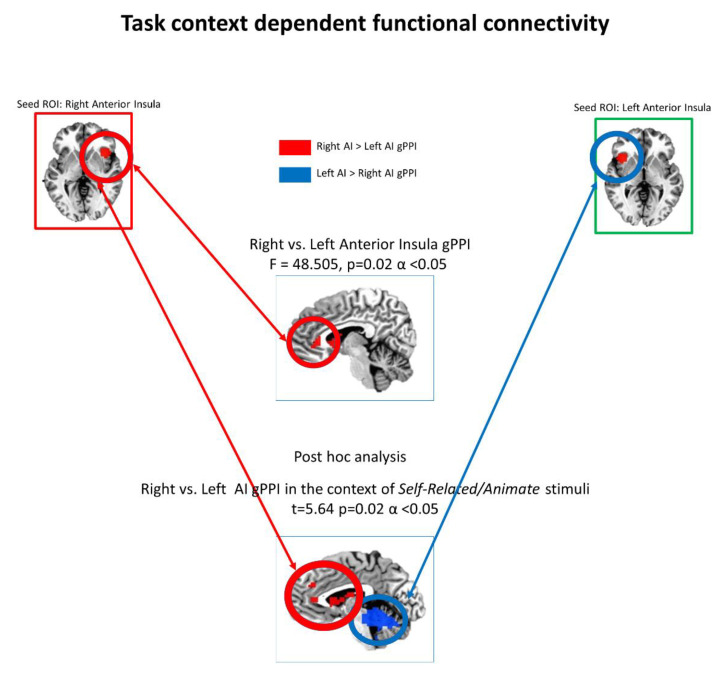
Task context-dependent functional connectivity—Generalized Psychophysiological Interaction—gPPI.

The generalized procedure of psychophysiological interactions [36] was used to calculate the condition-specific FC of regions of interest. According to our aim we used as seed regions for the gPPI the right anterior insula (MNI; *Cmass* x = 40, y = 19, z = −6) and the left anterior insula (MNI; *Cmass* x = −40, y = 19, z = −6) (see Figure 2). The gPPI procedure consisted in the following steps: (1) physiological seeds were obtained extracting the mean activity from non-zero voxels included in each ROI; (2) psychological variables for each regressor were created as vectors with the same temporal length as the seeds, and with a binary value of 1 or 0 for each TR, depending if the condition represented by the regressor was or was not active at that TR; (3) for each seed, extracted activity was deconvolved with a standard gamma function, and (4) multiplied with the psychological vector of each regressor; (5) each resulting vector, for each condition+, was re-convolved with the gamma function, and used as a PPI regressor. Nuisance regressors for motion, WM, and CSF were also included in the model. At the single subject level, a general linear model was performed for each ROI. At the group level, a whole brain measures 2 × 2 × 2 ANOVA with factors seed-ROI (levels: right and left anterior insula) target (levels: self-related and non self-related) and valence (levels: pleasant and unpleasant) was run in AFNI to study the context-dependent FC (βfc) of each ROI. Results were corrected via Monte Carlo simulation (using 3dClustSim), setting a threshold of *p* < 0.005 and a cluster size >40 voxels, to obtain a corrected significance level of α < 0.05. Post-hoc analysis focused predominantly on the comparison between right anterior insula and left anterior insula specifically for the self-related context-dependent FC in accordance with our hypotheses.

### 2.2. EEG

#### 2.2.1. EEG Sample

Twenty-seven participants were recruited. None of them had current or a history of psychiatric or neurological disorders and normal or corrected-to-normal vision. We excluded two participants due to technical problems while EEG recordings and one subject due to extreme results. Our final sample consists of 24 subjects (14 women, 10 men) with a mean age of 22.33 years (SD: 4.49, range: 19 to 36). Written informed consent was obtained. The study was approved by the local Ethics committee (REB # 2018054).

#### 2.2.2. Resting State

For the EEG recordings, participants performed 7 min of resting state with eyes open, fixing a screen with a black cross on a grey background.

#### 2.2.3. Stimuli

Our paradigm uses the well-known face-morphing technique [37,38,39,40,41]. Prior to the EEG recordings, pictures were taken from each of the participants with a Samsung A50 phone. Participants were asked to show a neutral facial expression, mouth closed. The pictures were then prepared in the free and open-source graphics editor GIMP (2.10.12) [42]. All faces were turned in black and white and cut in an oval circle, remaining only the eyes, nose and mouth areas on a black background. Then, each face was morphed with a same-sex and same-race face from the NimStim facial pictures set [43]. In addition, a same-sex famous face was morphed with an unknown face. Morphings were realized with the Abrosoft Fantamorph 5 Software [44] in 1% steps (from 0% to 100% self-face or 0% to 100% famous face).

#### 2.2.4. Morphing Task

Pictures were presented in a continuous manner to create the experience of a morphed movie between the two faces. A total of four morphing conditions were created based on the order and the identity depicted on the pictures: Self-conditions: (1) from ‘self’ to ‘other’ face, (2) from ‘other’ to ‘self’ face. Control conditions: (3) from ‘famous’ to ‘unknown’ and (4) from the ‘unknown’ to the ‘famous’ face. The order of presentation was randomized.

**Figure 3:**
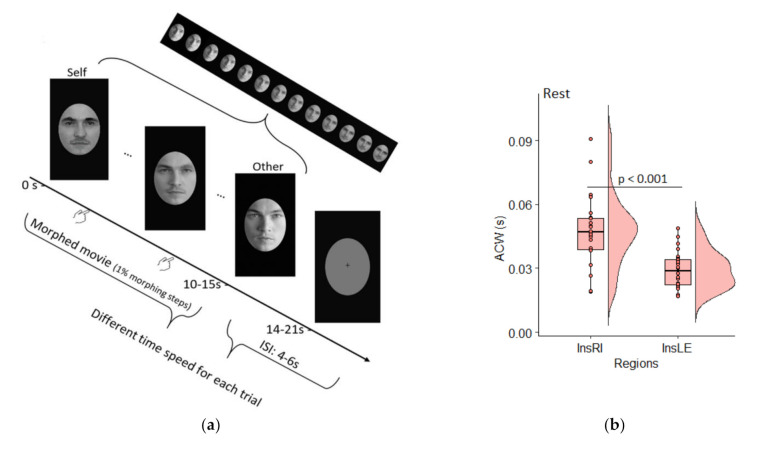

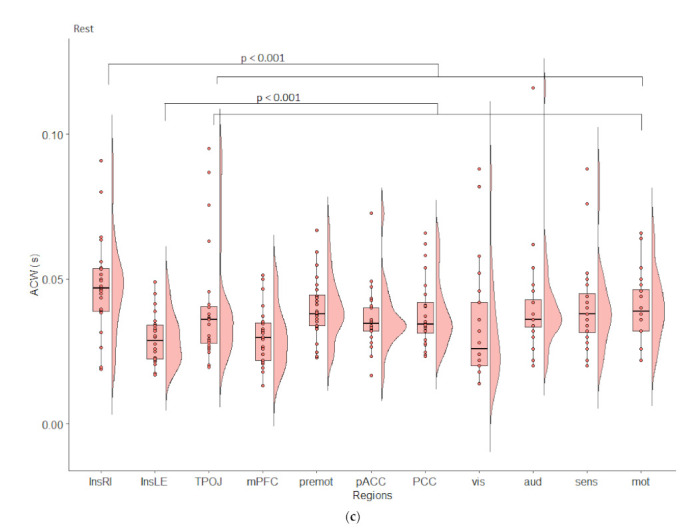
Autocorrelation calculated on the estimated activity per region in resting state. (**a**) Morphing task. Depicted is a schematic representation of the first condition ‘self to other’. Identity and order of faces vary depending on condition; (**b**) Longer ACW were found in the right Insula compared to the left insula; (**c**) The insula in comparison to primary sensory and motor regions as well as the regions implicated in the three layers model of the self. *p*-values are Bonferroni-corrected post hocs. Aud = primary auditory cortex, InsLE = left Insula, InsRI = right Insula, mot = primary motor cortex, mPFC = medial prefrontal cortex, pACC = perigenual anterior cingulate cortex, PCC = posterior cingulate cortex, premot = premotor cortex, sens = primary somatosensory cortex, TPOJ = temporo-parieto-occipital junction, vis = primary visual cortex.

Participants were instructed to press a key when they stop to see the first face and to give a second keypress when they started to see the second face. To ensure that the moment of the keypress was time-independent, we randomly varied the movie length between 10 to 15 s. In addition, the intertrial interval was jittered between 4 to 6 s (Figure 3a). In total there were 200 trials, 50 trials per condition in 8 blocks. But for the purpose of these analyses, we only used 7 min of continuous recording that were extracted from the second block. Also, we were interested in the general activity in task, therefore we do not distinguish between the conditions in future analyses.

#### 2.2.5. EEG Data Acquisition and Preprocessing

EEG data was recorded using Ag/AgCl electrodes through a 64-channel Brain Vision Easycap (according to the International 10–20 System) referenced to the right mastoid. The data was sampled at 1000 Hz with DC recording. The EEG data preprocessing was performed using the EEGLAB toolbox for MATLAB (R2017b; [45], RRID:SCR_007292). The data was downsampled to 500 Hz and filtered with a low-pass filter at 50 Hz and a high-pass filter at 1 Hz. With a customer script, noisy channels (defined as 4 interquartile above or below each channels mean) have been determined and spherically interpolated before re-referencing to the average. Further, artifacts were removed using independent component analysis (ICA) performed using the EEGLAB software creating 64 independent components. Next, we used MARA implementation to automatically reject noisy components [46].

#### 2.2.6. ROIs and Source Localization eLORETA 

**Figure 4:**
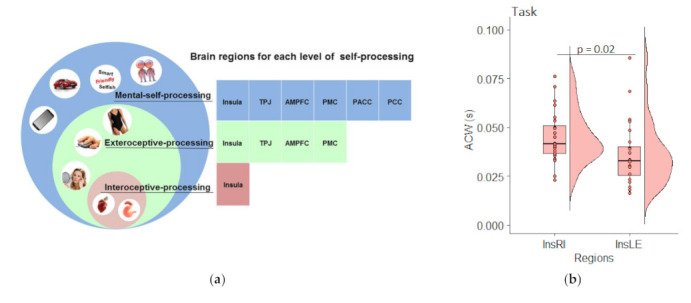

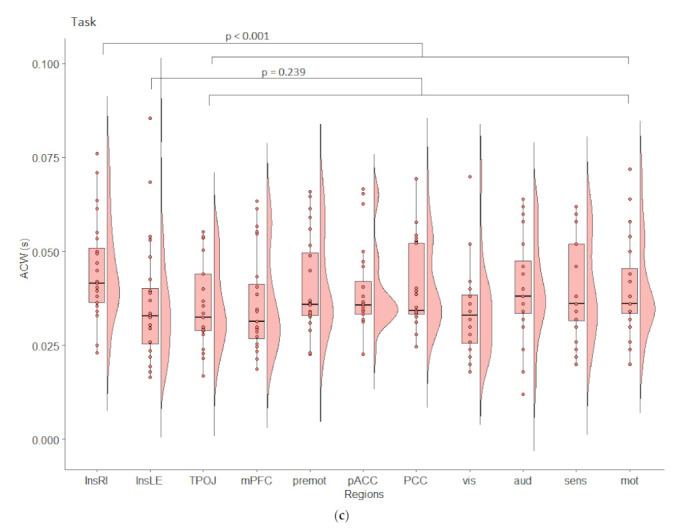
Autocorrelation calculated on the estimated activity per region in task state. (**a**) ROIs for the EEG study. Graph reproduced from Qin et al. (2020) with the kind permission of the authors; (**b**) Comparison of the left and right insula task state.; (**c**) Raincloud plots of ACW in task state in all ROIs. *p*-values represent Helmert contrasts. Aud = primary auditory cortex, InsLE = left Insula, InsRI = right Insula, mot = primary motor cortex, mPFC = medial prefrontal cortex, pACC = perigenual anterior cingulate cortex, PCC = posterior cingulate cortex, premot = premotor cortex, sens = primary somatosensory cortex, TPOJ = temporo-parieto-occipital junction, vis = primary visual cortex.

We defined our ROIs for the EEG analysis based on six main regions implicated in the three layers of the self as emphasied in the meta-analysis of Qin et al. [17] (Figure 4a). These regions are the insula, the temporo-parieto-occipital junction (TPOJ), the anteromedial prefrontal cortex (amPFC), the premotor cortex (PMC), the perigenual anterior cingulate cortex (pACC) and the posterior cingulate cortex (PCC). As a control, we also included the primary motor, primary visual, primary auditory and primary somatosensory cortex. Regions were defined according to the Glasser parcellations [47].

Exact Low Resolution Electromagnetic Tomography (eLORETA, [48]) was performed by estimating the virtual-channel source for each region of the Glasser atlas in FieldTrip [49]. The output is a timeseries of estimated source level activity for each region. We investigated the right and left insula separately while averaging the right and left parts of the other regions (See Supplementary Table S1). On these timeseries, we calculated ACW.

#### 2.2.7. Autocorrelation Window 

The autocorrelation window (ACW) is defined as full-width-at-half-maximum of the temporal autocorrelation function of a time series and plays an important role in information integration [50,51]. It was calculated in python 3 using the autocorrelation function with the statmodels package on the regional activity extracted via eLORETA. The ACW is in units of s, that is to say, an ACW of 0.06 s indicates that the correlation between the estimated timeseries with itself is correlated with an r = 0.5 at a temporal shift of 0.06 s.

#### 2.2.8. Statistical Analysis

Due to multiple measures per subject on different brain regions in two different states (rest and task state), we computed repeated measures ANOVA with a Greenhouse-Geisser correction when sphericity was violated. Helmert contrasts for either the right or left insula against all other regions, in resting as well as in task state, mark the special role of the insula. Analyses were run in R and JASP.

## 3. Results

### 3.1. Resting State fMRI Analysis

In order to estimate the centrality of the insula in the spontaneous activity of the brain, we applied a network analysis approach using as nodes of each network the ROIs related to the three different layers of self, as recently proposed by [17], i.e., (a) interoceptive self, (b) extero-ceptive self, (c) mental self (See Figure 1a–c).

For the intero-ceptive self network as shown in Figure 1a, we can observe that the nodes showing stronger *Degree (nodal strength)* are the dorsal anterior cingulate cortex, the thalamus, the left insula, and the superior temporal gyrus. The stronger *Betweenness* is represented by the thalamus while the stronger *Closeness* is represented by both right and left anterior insula.

For the extero- proprio-ceptive self network as shown in Figure 1b, we can observe that the nodes showing stronger *Degree (nodal strength)* are right insula, inferior parietal lobule, and the medial prefrontal cortex. The stronger *Betweenness* and *Closeness* is represented by superior parietal lobule, inferior parietal lobule and medial prefrontal cortex. As it can be observed in this layer of self, right insula showed a significant higher degree of centrality in comparison with left insula.

For the mental self network as shown in Figure 1c, we can observe the central role of right insula together with posterior cingulate cortex for all three measures *Degree (nodal strength), Betweenness* and *Closeness*. As it can be observed left insula did not show any significant centrality role within this network.

Altogether these findings show the increasing “integrative role” (higher centrality metrics reflect how connected and potentially relevant a node is within a network) of the right insula over the increased hierarchical layer of self: (a) the right insula shows a significant closeness centrality role (i.e., the average distance of a node to all the other nodes in the network) together with the left insula (showing also higher DC) in the interoceptive layer of self; (b) the right insula shows a strong degree (nodal strength) in the exteroceptive layer of self (i.e., the sum of the absolute value of the edge weights) and (c) the right insula has a prominent role in all the three measures of centrality (degree, closeness and betweenness) in the mental layer of self while the left anterior insula does not show a central role in the network. To put it simple, the centrality of the right vs. the left insula already in resting state fMRI increases together with the hierarchical increase of self-processing showing the most prominent role within the mental self-network.

### 3.2. Task Context-Dependent Functional Connectivity

To evaluate the context-dependent connectivity (self-related context and non self-related context), gPPI was performed using as seed a right and a left anterior insula regions of interests. This allowed to identify voxel clusters whose functional connectivity with the seeds ROIs was significantly modulated by the different contexts during the task. At a group level no significant effects were found for the factor valence, and for the interaction between target and valence for both seed ROIs. Thus, we focused our investigation on seed ROI level (right and left anterior insula) and on the target level (self-related context and non self-related context). A repeated measures ANOVA showed highly significant differences between seed ROIs (F = 48.505, *p* = 0.02, α < 0.05) indicating that the right anterior insula is more connected with pACC. Post-hoc analysis revealed a significant difference (t = 5.64; *p* = 0.02, α < 0.05) between right and left anterior insula FC during the context of self-related processing with right AI > left AI for regions like PACC (MNI x = 8, y = −41; z = −9) and right caudate nucleus, middle right insula (MNI x = 40 y = 11; z = −13) and left middle temporal gyrus while left > right in regions like left cerebellum cortex (MNI x-40, y-58 z-32). No significant difference with the same threshold were found between right and left insula for the non self-related context (See Figure 2). 

In sum these gPPI findings show the increased context dependent FC between Right Anterior Insula (but not Left AI) and typical self regions like pACC during self-related processing (vs. non self-related). This confirms the functional integrative role of the right AI in self-specific task.

### 3.3. Temporal Analyses in EEG

In order to estimate the length of the INTs in the insula, we compared the ACW on the estimated activity in the left and right Insula in resting state (Figure 3b). Results indicate a highly significant difference (t(24) = 5.21, *p* < 0.001) with a longer ACW in the right (m = 0.048, SD = 0.017) compared to the left insula (m = 0.029, SD = 0.009).

Does this difference in the INT remain in comparison to other self-related and unisensory areas? A repeated measures ANOVA showed highly significant differences between regions (F(4.7, 108.21) = 5.26, *p* = 0.0003). A Helmert contrast revealed significant differences in ACW for the right (*p* < 0.001) and left (*p* < 0.001) insula compared to the self and unisensory regions (Figure 3c). Interestingly, the left insula is significantly shorter than the other self- and unisensory regions. This confirms our hypothesis of longer INT in the right insula in resting state. Does this difference between right and left insula maintain in a self-task? And does the right insula maintain the longer INT compared to the other regions in the task?

To answer this question, we ran a repeated measures ANOVA between left and right insula in task and can confirm a significant difference between these two regions (F (1, 23) = 6.224, *p* = 0.020; Figure 4b). Next, we investigated the left and right insula in the context of the other regions (Figure 4c) and found a significant effect of the region (F (1.07, 24.65) = 64.327, *p* < 0.001). Helmert contrasts confirm that the right (*p* < 0.001) but not the left insula (*p* = 0.239) is significantly longer compared to the unisensory or self [17] regions. This confirms the special role of the right insula.

## 4. Discussion

We here investigated the neural topography and dynamic of right and left insula with respect to those regions implicated in the different layers of self. Our main findings are: (i) significant higher degree (nodal strength and other network-related measures) of the right anterior insula than left insula over the hierarchical layers of self; (ii) higher context dependent functional connectivity between right anterior insula (vs. left) and other regions implicated in the context of self-related processing; (iii) significantly longer temporal windows, i.e., autocorrelation window, in the neural activity of the right anterior insula than the left anterior insula during both rest and self-specific task; (iv) significantly longer autocorrelation window during a self-specific task in right anterior insula compared to the other regions implicated in the three layers of self. Together, our findings show special topographic and dynamic features in the neural activity of the right insula compared to both left insula and other self-specific regions. This renders neural activity in the right insula highly suitable to serve as topographic and dynamic glue or node for integrating different layers of self.

### 4.1. From Functional Connectivity over Functional Integration to Spatial Nestedness of Self

Using resting state fMRI, we show that the right anterior insula exhibits increasing centrality indices over the three hierarchical layers of self in comparison with all other regions implicated. Our findings extend previous imaging studies that demonstrate strong recruitment of the right anterior insula during self-referential tasks [6,7,8,9,10,11,14,17,52]. These studies usually demonstrate the conjoint recruitment of right/left anterior insula with the cortical midline structures (CMS) and potentially other regions implicated in self. Our findings provide the topographic substrate of such conjoint activation of right (and left) insula and CMS: this, as we propose, is due to the increasing centrality and functional connectivity of the right insula to other regions like the CMN over the hierarchical self-networks. Intriguingly our findings don’t deny the role of the left insula, which showed significant centrality indices especially for the intero-ceptive self processing network. This might be in line with the fact that many studies on self-referential tasks [6,7,8,9,10,11,14,17,52] usually demonstrate the conjoint recruitment of right and left anterior insula together with the CMS.

High centrality and functional connectivity mean that the right insula integrates the activity of the other regions of the self-networks. Functional connectivity allows for functional integration, that is, the degree to which a region pools or sums the activity of other regions within its own neural activity [53,54]. The high degree of functional integration enables the right insula to integrate interoceptive, exteroceptive, proprioceptive, and cognitive/mental information from the three layers of the self-networks. That, in turn, allows for an intrinsic link or connection between the three layers of self-specific information which are thereby spatially or topographically nested within each other [17]. We hypothesize that the right insula’s key role in constituting spatial nestedness on the neuronal level, i.e., among the three layers of self-networks may also be manifest on the psychological level: self-specific interoceptive information may be contained and nested within the layer of self-specific proprio- and exteroceptive information which, in turn, may be nested and contained within the even more extended layer of self-specific mental or cognitive information. That remains to be investigated though. If true, spatial nestedness may provide a shared feature of neural and mental levels of self, i.e., their “common currency” [20,21].

### 4.2. From Autocorrelation Window over Temporal Integration to Temporal Continuity of Self

Our second main finding consists in the observation of longer INT in the right insula compared to the left insula and the other regions implicated in the three layers of self. This was already present during the resting state and carried over to the task states during the processing of self-specific information, i.e., the own face. The involvement of longer INT in right insula complements recent resting state findings that show a relationship of ACW and self-consciousness: the longer the ACW in the resting state, the higher degree of self-consciousness [55] and the better self-specificity of information is integrated and preserved across different temporal delays [56].

INT are key in the temporal integration and segregation of inputs at different points in time [22,23,24]. The longer the time windows of the neural activity of a region, the more inputs at different even distant points in time can be lumped and pooled together under the umbrella of one temporal window and subsequently elicit one (rather than multiple) neural activity change, i.e., task-related activity [57]. The right insula’s longer ACW may thus suit ideally to integrate, i.e., pool and sum different inputs occurring at different points in time: the different time points of intero-, extero, and proprioceptive and cognitive/mental inputs are subsumed within one temporal window on the basis which they induce one integrated (rather than two or several parallel) activity change within the right insula.

**Figure 5:**
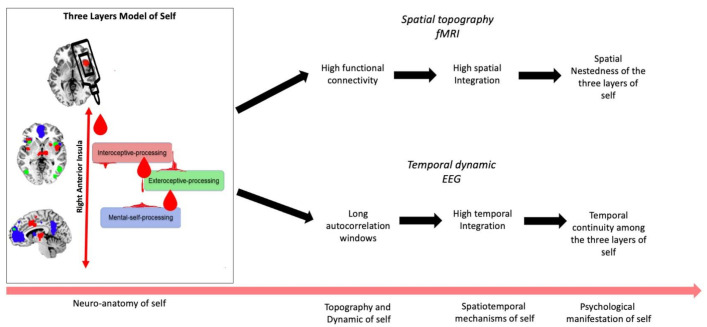
Visual representation of spatial and temporal mechanisms of the three layers of self. The left part of the figure shows that the glue (drops) provided by the right anterior insula joins all the networks, e.g., interoceptive- exteroceptive- and mental-self processing in accordance with Qin et al. 2020. The right side of the figure highlights the key role of right anterior insula for functional/spatial and temporal integration in our fMRI and EEG findings. Overall, this figure represents how neuronal data might translate in spatio-temporal mechanism and psychological manifestation of self.

How is such high degree of temporal integration on the neural level manifest on the psychological level of self? Neuronally, different inputs at distinct time points are integrated within the neural activity of the right insula and the regions of the three layers of self-specific networks. Temporal integration of different time points’ inputs in neural activity means that those temporally distinct inputs are connected to each other within the duration of the integrating temporal window. This constitutes temporal continuity between the originally temporally distinct inputs. The long time windows of right insula compared to both left insula and other self-specific regions suit it ideally for constituting a high degree of temporal continuity among the three distinct layers of self, i.e., interoceptive, extero-proprioceptive, and mental/cognitive. Such temporal continuity may amount to what is described by the concept of personal identity [58,59]. Temporal continuity may thus provide a shared feature of neural and psychological levels of self/personal identity, i.e., their “common currency” [20,21]. Figure 5 resumes the roles of the right insula (glue between the three layers of the self) with its spatial (upper row) and temporal (lower row) processes.

### 4.3. Methodological Limitations

One limitation is that we did not recruit the same subjects for fMRI and EEG. Moreover, different paradigms like self-related/animate vs. non-self-related/inanimate and self-enfacement were run in fMRI and EEG respectively. While this is clearly a limitation, it can also be taken as litmus test for the validity of our findings. Despite different paradigms and different subjects, both modalities show analogous converging findings, the topographic and dynamic specialness of right insula compared to left insula.

Yet another limitation is that we did not go into detail about the neuro-anatomical differentiations within the insula itself. This concerns anterior and posterior as well as dorsal and ventral parts of the insula [60,61,62]. Instead, we here focused only on mainly the anterior insula as this is the region of interest we obtained from the Qin et al. [17] meta-analyses. Particularly in EEG is to consider that the insula is a deeply located structure and the recorded activity may be contaminated by the temporal and frontoparietal opercula.

One can also argue that we here focused only on those regions implicated in the three layers of the self-networks defined by Qin et al. [17]. This carries the advantage that we can really make specific assumption of the right insula with respect to specifically the three layers of the self-networks. While the disadvantage consists in the fact that we cannot make any assumptions about the particular role of the right insula for the brain’s global activity and its topography [63]. That was not our aim, though, as our focus was mainly on the special role of the right insula for the self. Moreover, another limitation is represented by the fact that those *a priori* defined regions are dysbalanced in terms of laterality. For instance, for the exteroceptive-self layer 9 regions on 14 (64%) are located on the right side of the brain while for the mental-self only 3 regions on 12 (25%) are located at the right side of the brain. However, in both cases right anterior insula showed higher centrality indices when compared to the left one. Considering the intrinsic limitation represented by the laterality disbalance and the different number of regions/nodes for each network that might affect the analysis, this seems to tentatively suggest that nor the prevalence of right regions in exteroceptive-self processing network nor the prevalence of left regions in mental-self processing network affect the role of right insula within the networks.

Furthermore, the fMRI sample is composed only by men. This limits the generalizability of our findings and leave open the question whether there are functional brain differences between males and females across the different networks. Future research needs to confirm these data on other samples including both men and women to further support our interpretations and extend the generalizability of our findings.

Finally, we did not directly investigate familiarity. We can only infer from the Qin et al. study that the key difference between self-specificity and familiarity consists in the absence of the right/left insula in familiarity. One major psychological difference between familiarity and self-specificity is that only the latter but not the former involves the own body including intero- and proprioceptive feelings/sensations. Given its role in interoceptive and proprioceptive processing as well as intero-exteroceptive integration [15,16,64,65,66], the right insula may be key in yielding a particular feeling or subjective experience, a sense of self, whereas such feeling is not present in familiarity.

## 5. Conclusions 

What renders neural activity in the right rather than left anterior insula more suitable to take on a special role in mediating self-specificity? We here show that neural activity in the right insula exhibits special topographic and dynamic features compared to the left insula. The right insula shows higher degree of functional integration with respect to other regions implicated in the different layers of self. Moreover, the right insula exhibits longer time windows in its neural activity than both the left insula and the other regions of the different layers of self. Together, these findings suggest higher degrees of both functional and temporal integration in the neural activity of the right insula. This suits the right insula ideally to serve as topographic and dynamic node or glue between the distinct layers of self ensuring their high degrees of spatial nestedness and temporal continuity.

## Data Availability

The data are not publicly available due to restrictions e.g. their containing information that could compromise the privacy of research participants. The data that support the findings of this study can be requested to the corresponding author.

## Supplementary Materials

The following are available online at https://www.mdpi.com/article/10.3390/brainsci11101312/s1, Table S1: Composition of ROIs for the EEG analyses. ACW was calculated on each region individually and then average to build the ROIs.

## References

[1] Sui J., Humphreys G. (2015). The Integrative Self: How Self-Reference Integrates Perception and Memory. Trends Cogn. Sci..

[2] Northoff G. (2016). Is the Self a Higher-Order or Fundamental Function of the Brain? The “Basis Model of Self-Specificity” and Its Encoding by the Brain’s Spontaneous Activity. Cogn. Neurosci..

[3] Northoff G., Bermpohl F. (2004). Cortical Midline Structures and the Self. Trends Cogn. Sci..

[4] Frewen P., Schroeter M.L., Riva G., Cipresso P., Fairfield B., Padulo C., Kemp A.H., Palaniyappan L., Owolabi M., Kusi-Mensah K. (2020). Neuroimaging the Consciousness of Self: Review, and Conceptual-Methodological Framework. Neurosci. Biobehav. Rev..

[5] Davey C.G., Pujol J., Harrison B.J. (2016). Mapping the Self in the Brain’s Default Mode Network. NeuroImage.

[6] Murray R.J., Schaer M., Debbané M. (2012). Degrees of Separation: A Quantitative Neuroimaging Meta-Analysis Investigating Self-Specificity and Shared Neural Activation between Self- and Other-Reflection. Neurosci. Biobehav. Rev..

[7] Modinos G., Ormel J., Aleman A. (2009). Activation of Anterior Insula during Self-Reflection. PLoS ONE.

[8] Scalabrini A., Ebisch S.J.H., Huang Z., Di Plinio S., Perrucci M.G., Romani G.L., Mucci C., Northoff G. (2019). Spontaneous Brain Activity Predicts Task-Evoked Activity During Animate Versus Inanimate Touch. Cereb. Cortex.

[9] Scalabrini A., Huang Z., Mucci C., Perrucci M.G., Ferretti A., Fossati A., Romani G.L., Northoff G., Ebisch S.J.H. (2017). How Spontaneous Brain Activity and Narcissistic Features Shape Social Interaction. Sci. Rep..

[10] Qin P., Northoff G. (2011). How Is Our Self Related to Midline Regions and the Default-Mode Network?. NeuroImage.

[11] Enzi B., de Greck M., Prösch U., Tempelmann C., Northoff G. (2009). Is Our Self Nothing but Reward? Neuronal Overlap and Distinction between Reward and Personal Relevance and Its Relation to Human Personality. PLoS ONE.

[12] Babo-Rebelo M., Wolpert N., Adam C., Hasboun D., Tallon-Baudry C. (2016). Is the Cardiac Monitoring Function Related to the Self in Both the Default Network and Right Anterior Insula?. Philos. Trans. R. Soc. B Biol. Sci..

[13] Fan Y., Duncan N.W., de Greck M., Northoff G. (2011). Is There a Core Neural Network in Empathy? An FMRI Based Quantitative Meta-Analysis. Neurosci. Biobehav. Rev..

[14] D’Argembeau A., Collette F., Van der Linden M., Laureys S., Del Fiore G., Degueldre C., Luxen A., Salmon E. (2005). Self-Referential Reflective Activity and Its Relationship with Rest: A PET Study. NeuroImage.

[15] Craig A.D. (2003). Interoception: The Sense of the Physiological Condition of the Body. Curr. Opin. Neurobiol..

[16] Craig A.D. (2010). (Bud) The Sentient Self. Brain Struct. Funct..

[17] Qin P., Wang M., Northoff G. (2020). Linking Bodily, Environmental and Mental States in the Self—A Three-Level Model Based on a Meta-Analysis. Neurosci. Biobehav. Rev..

[18] Blanke O., Slater M., Serino A. (2015). Behavioral, Neural, and Computational Principles of Bodily Self-Consciousness. Neuron.

[19] Scalabrini A., Xu J., Northoff G. (2021). What COVID -19 Tells Us about the Self: The Deep Intersubjective and Cultural Layers of Our Brain. Psychiatry Clin. Neurosci..

[20] Northoff G., Wainio-Theberge S., Evers K. (2020). Spatiotemporal Neuroscience–What Is It and Why We Need It. Phys. Life Rev..

[21] Northoff G., Wainio-Theberge S., Evers K. (2020). Is Temporo-Spatial Dynamics the “Common Currency” of Brain and Mind? In Quest of “Spatiotemporal Neuroscience.”. Phys. Life Rev..

[22] Golesorkhi M., Gomez-Pilar J., Tumati S., Fraser M., Northoff G. (2021). Temporal Hierarchy of Intrinsic Neural Timescales Converges with Spatial Core-Periphery Organization. Commun. Biol..

[23] Golesorkhi M., Gomez-Pilar J., Zilio F., Berberian N., Wolff A., Yagoub M.C.E., Northoff G. (2021). The Brain and Its Time: Intrinsic Neural Timescales Are Key for Input Processing. Commun. Biol..

[24] Zilio F., Gomez-Pilar J., Cao S., Zhang J., Zang D., Qi Z., Tan J., Hiromi T., Wu X., Fogel S. (2021). Are Intrinsic Neural Timescales Related to Sensory Processing? Evidence from Abnormal Behavioral States. NeuroImage.

[25] Cox R.W. (1996). AFNI: Software for Analysis and Visualization of Functional Magnetic Resonance Neuroimages. Comput. Biomed. Res..

[26] Johnstone T., Walsh K.S.O., Greischar L.L., Alexander A.L., Fox A.S., Davidson R.J., Oakes T.R. (2006). Motion Correction and the Use of Motion Covariates in Multiple-subject FMRI Analysis. Hum. Brain. Mapp..

[27] Fox M.D., Snyder A.Z., Vincent J.L., Corbetta M., Raichle M.E. (2005). The Human Brain Is Intrinsically Organized into Dynamic, Anticorrelated Functional Networks. Proc. Natl. Acad. Sci. USA.

[28] Chai X.J., Castañón A.N., Öngür D., Whitfield-Gabrieli S. (2012). Anticorrelations in Resting State Networks without Global Signal Regression. NeuroImage.

[29] Van Dijk K.R.A., Hedden T., Venkataraman A., Evans K.C., Lazar S.W., Buckner R.L. (2010). Intrinsic Functional Connectivity As a Tool For Human Connectomics: Theory, Properties, and Optimization. J. Neurophsyiology.

[30] He B.J. (2011). Scale-Free Properties of the Functional Magnetic Resonance Imaging Signal during Rest and Task. J. Neurosci..

[31] Murphy K., Fox M.D. (2017). Towards a Consensus Regarding Global Signal Regression for Resting State Functional Connectivity MRI. NeuroImage.

[32] Scalabrini A., Vai B., Poletti S., Damiani S., Mucci C., Colombo C., Zanardi R., Benedetti F., Northoff G. (2020). All Roads Lead to the Default-Mode Network—Global Source of DMN Abnormalities in Major Depressive Disorder. Neuropsychopharmacology.

[33] Friedman J., Hastie T., Tibshirani R. (2008). Sparse Inverse Covariance Estimation with the Graphical Lasso. Biostatistics.

[34] Epskamp S., Borsboom D., Fried E.I. (2018). Estimating Psychological Networks and Their Accuracy: A Tutorial Paper. Behav. Res. Methods.

[35] Epskamp S., Cramer A.O.J., Waldorp L.J., Schmittmann V.D., Borsboom D. (2012). Qgraph: Network Visualizations of Relationships in Psychometric Data. J. Stat. Softw..

[36] McLaren D.G., Ries M.L., Xu G., Johnson S.C. (2012). A Generalized Form of Context-Dependent Psychophysiological Interactions (GPPI): A Comparison to Standard Approaches. NeuroImage.

[37] Keenan J.P., Freund S., Hamilton R.H., Ganis G., Pascual-Leone A. (2000). Hand Response Differences in a Self-Face Identification Task. Neuropsychologia.

[38] Heuer K., Lange W.-G., Isaac L., Rinck M., Becker E.S. (2010). Morphed Emotional Faces: Emotion Detection and Misinterpretation in Social Anxiety. J. Behav. Ther. Exp. Psychiatry.

[39] Campanella S., Hanoteau C., Depy D., Rossion B., Bruyer R., Crommelinck M., Guerit J.M. (2000). Right N170 Modulation in a Face Discrimination Task: An Account for Categorical Perception of Familiar Faces. Psychophysiology.

[40] Sandsten K.E., Nordgaard J., Kjaer T.W., Gallese V., Ardizzi M., Ferroni F., Petersen J., Parnas J. (2020). Altered Self-Recognition in Patients with Schizophrenia. Schizophr. Res..

[41] Miller J.G., Shrestha S., Reiss A.L., Vrtička P. (2020). Neural Bases of Social Feedback Processing and Self–Other Distinction in Late Childhood: The Role of Attachment and Age. Cogn. Affect. Behav. Neurosci..

[42] Wilber GIMP-GIMP 2.10.12 Released. https://www.gimp.org/news/2019/06/12/gimp-2-10-12-released/.

[43] Tottenham N., Tanaka J.W., Leon A.C., McCarry T., Nurse M., Hare T.A., Marcus D.J., Westerlund A., Casey B., Nelson C. (2009). The NimStim Set of Facial Expressions: Judgments from Untrained Research Participants. Psychiatry Res..

[44] Abrosoft FantaMorph-Photo Morphing Software for Creating Morphing Photos and Animations. https://www.fantamorph.com/.

[45] Delorme A., Makeig S. (2004). EEGLAB: An Open Source Toolbox for Analysis of Single-Trial EEG Dynamics Including Independent Component Analysis. J. Neurosci. Methods.

[46] Winkler I., Haufe S., Tangermann M. (2011). Automatic Classification of Artifactual ICA-Components for Artifact Removal in EEG Signals. Behav. Brain Funct..

[47] Glasser M.F., Coalson T.S., Robinson E.C., Hacker C.D., Harwell J., Yacoub E., Ugurbil K., Andersson J., Beckmann C.F., Jenkinson M. (2016). A Multi-Modal Parcellation of Human Cerebral Cortex. Nature.

[48] Pascual-Marqui R.D., Michel C.M., Lehmann D. (1994). Low Resolution Electromagnetic Tomography: A New Method for Localizing Electrical Activity in the Brain. Int. J. Psychophysiol..

[49] Oostenveld R., Fries P., Maris E., Schoffelen J.-M. (2011). FieldTrip: Open Source Software for Advanced Analysis of MEG, EEG, and Invasive Electrophysiological Data. Comput. Intell. Neurosci..

[50] Murray J., Bernacchia A., Freedman D., Romo R., Wallis J., Cai X., Padoa Schioppa C., Pasternak T., Seo H., Lee D. (2014). A Hierarchy of Intrinsic Timescales across Primate Cortex. Nat. Neurosci..

[51] Honey C.J., Thesen T., Donner T.H., Silbert L.J., Carlson C.E., Devinsky O., Doyle W.K., Rubin N., Heeger D.J., Hasson U. (2012). Slow Cortical Dynamics and the Accumulation of Information over Long Timescales. Neuron.

[52] Murray R.J., Debbané M., Fox P.T., Bzdok D., Eickhoff S.B. (2015). Functional Connectivity Mapping of Regions Associated with Self- and Other-Processing. Hum. Brain Mapp..

[53] Deco G., Tononi G., Boly M., Kringelbach M.L. (2015). Rethinking Segregation and Integration: Contributions of Whole-Brain Modelling. Nat. Rev. Neurosci..

[54] Scalabrini A., Mucci C., Esposito R., Damiani S., Northoff G. (2020). Dissociation as a Disorder of Integration–On the Footsteps of Pierre Janet. Prog. Neuropsychopharmacol. Biol. Psychiatry.

[55] Wolff A., Di Giovanni D.A., Gómez-Pilar J., Nakao T., Huang Z., Longtin A., Northoff G. (2019). The Temporal Signature of Self: Temporal Measures of Resting-State EEG Predict Self-Consciousness. Hum. Brain Mapp..

[56] Kolvoort I.R., Wainio-Theberge S., Wolff A., Northoff G. (2020). Temporal Integration as “Common Currency” of Brain and Self-Scale-Free Activity in Resting-State EEG Correlates with Temporal Delay Effects on Self-Relatedness. Hum. Brain Mapp..

[57] Himberger K.D., Chien H.-Y., Honey C.J. (2018). Principles of Temporal Processing Across the Cortical Hierarchy. Neuroscience.

[58] Ersner-Hershfield H., Wimmer G.E., Knutson B. (2009). Saving for the Future Self: Neural Measures of Future Self-Continuity Predict Temporal Discounting. Soc. Cogn. Affect. Neurosci..

[59] Northoff G., Magioncalda P., Martino M., Lee H.-C., Tseng Y.-C., Lane T. (2018). Too Fast or Too Slow? Time and Neuronal Variability in Bipolar Disorder—A Combined Theoretical and Empirical Investigation. Schizophr. Bull..

[60] (Bud) Craig A.D. (2011). Significance of the Insula for the Evolution of Human Awareness of Feelings from the Body. Ann. N. Y. Acad. Sci..

[61] (Bud) Craig A.D. (2009). How Do You Feel—Now? The Anterior Insula and Human Awareness. Nat. Rev. Neurosci..

[62] Uddin L.Q., Nomi J.S., Hébert-Seropian B., Ghaziri J., Boucher O. (2017). Structure and Function of the Human Insula. J. Clin. Neurophysiol..

[63] Zhang J., Huang Z., Tumati S., Northoff G. (2020). Rest-Task Modulation of FMRI-Derived Global Signal Topography Is Mediated by Transient Coactivation Patterns. PLoS Biol..

[64] Wiebking C., Northoff G. (2014). Interoceptive Awareness and the Insula–Application of Neuroimaging Techniques in Psychotherapy. GSTF J. Psychol. JPsych.

[65] Wiebking C., Duncan N.W., Tiret B., Hayes D.J., Marjaǹska M., Doyon J., Bajbouj M., Northoff G. (2014). GABA in the Insula—A Predictor of the Neural Response to Interoceptive Awareness. NeuroImage.

[66] Wiebking C., de Greck M., Duncan N.W., Tempelmann C., Bajbouj M., Northoff G. (2015). Interoception in Insula Subregions as a Possible State Marker for Depression—an Exploratory FMRI Study Investigating Healthy, Depressed and Remitted Participants. Front. Behav. Neurosci..

