# Supplementary material for: The Self and Its Right Insula—Differential Topography and Dynamic of Right vs. Left Insula
Source: Brain Sci. 2021 Oct 2;11(10):1312. doi: 10.3390/brainsci11101312 (PMC8533814; doi:10.3390/brainsci11101312)
Supplement: Supplementary file 1 [file brainsci-11-01312-s001.zip › brainsci-1383272-supplementary.pdf]

Supplementary

Table S1. Composition of ROIs for the EEG analyses. ACW was calculated on each region individually and then average to build the ROIs.

ROI	Subregions
InsRI	R_RI,R_PoI2,R_MI,R_AVI, R_AAIC, R_PoI1, R_Ig,R_PI
InsLE	L_RI, L_PoI2, L_MI, L_AVI, L_AAIC, L_PoI1, L_Ig, L_PI
PCC	L_DVT, R_DVT, L_ProS, R_ProS, L_POS1, R_POS1, L_POS2, R_POS2, L_RSC, R_RSC, L_v23ab, R_v23ab, L_23d, R_23d, L_d23ab, R_d23ab, L_23c, R_23c, L_PCV, R_PCV, L_7m, R_7m, L_31pd, R_31pd, L_31a, R_31a, L_31pv, R_31pv
pACC	R_p24, L_p24, R_a24, L_a24, R_p32pr, L_p32pr
Premotor cortex	R_6d, L_6d, R_FEF, L_FEF,R_55b, L_55b,R_6v, L_6v, R_6r, L_6r,R_PEF, L_PEF
TPOJ	R_TPOJ1, L_TPOJ1, R_TPOJ2, L_TPOJ2,R_TPOJ3, L_TPOJ3,R_STV, L_STV, R_PSL, L_PSL
mPFC	R_9m, L_9m, R_10d, L_10d,R_9a, L_9a
Primary visual	L_V1, R_V1
Primary auditive	L_A1, R_A1
Primary motor	L_4, R_4
Primary sensorymotor	L_3b, R_3b
